# Thyroid autoimmunity and spontaneous cervicocranial artery dissection: an exploratory segment-specific case-control study

**DOI:** 10.3389/fcvm.2026.1733516

**Published:** 2026-07-15

**Authors:** Yaqiong Yang, Zhenxiang Zhao, Ningning Che, Wei Mo

**Affiliations:** 1 Department of Neurology, The First Affiliated Hospital of Zhengzhou University, Zhengzhou, Henan, China; 2 Department of Neurology, Henan Provincial People’s Hospital, Zhengzhou, Henan, China; 3 Henan University of Chinese Medicine Henan Provincial Hospital of TCM, The Second Affiliated Hospital of Henan University of Chinese Medicine, Zhengzhou, Henan, China

**Keywords:** cervicocranial artery dissection, TGAb, thyroid autoantibody, thyroid autoimmunity, TPOAb

## Abstract

**Introduction:**

Immune-mediated mechanisms are thought to contribute to the local vascular inflammation associated with spontaneous cervicocranial arterial dissection (SCCAD). However, evidence linking thyroid autoimmunity to SCCAD remains conflicting. Furthermore, the potential correlation between thyroid autoimmunity and the anatomical distribution of dissections has not been evaluated.

**Methods:**

This retrospective, hospital-based case-control study was conducted from June 2021 to May 2025, enrolling 164 patients with SCCAD and 164 healthy controls. Multivariate logistic regression analyses, and patient-level sensitivity analyses were employed to examine the impact of thyroid autoantibodies on SCCAD and their anatomical distributions. Additionally, propensity score matching, stratified analyses and interaction tests were performed to assess the robustness of the association between thyroid autoimmunity and SCCAD.

**Results:**

Multivariate analyses showed that, compared with healthy controls, SCCAD had significantly higher rates of thyroid autoimmunity positivity (OR 6.715; 95% CI: 1.589–28.377; *P* = 0.010) and elevated TPOAb levels (OR 5.999; 95% CI: 1.379–26.096; *P* = 0.017). In segment-specific analyses, elevated TPOAb levels were positively associated with V4 segment involvement (OR 7.663, 95% CI: 1.443–40.680, *P* = 0.017) and inversely associated with V3 segment involvement (OR 0.065, 95% CI: 0.005–0.816, *P* = 0.034). The robustness of these segment-specific associations was further supported by patient-level sensitivity analyses (V3: OR 0.043, 95% CI: 0.003–0.669, *P* = 0.025; V4: OR 10.452, 95% CI: 1.143–95.584, *P* = 0.038).

**Discussion:**

In conclusion, thyroid autoimmunity positivity and elevated TPOAb were independently associated with SCCAD, and TPOAb demonstrated a previously unrecognized segment-specific association with vertebral artery dissections—positive at V4 and inverse at V3.

## Introduction

Although the etiology and pathogenesis of spontaneous cervicocranial arterial dissection (SCCAD) are not fully understood, local inflammation and autoimmunity are implicated. Mounting evidence supports the involvement of proinflammatory and immune-mediated mechanisms in the cascade of events leading to SCCAD. This includes observations of elevated leukocytes and C-reactive protein (1, 2), imaging data suggesting local inflammation in dissected vessels (3), histopathological findings of inflammatory infiltrates around coronary arteries (4), and vasculitis-like presentations in dissection cases (5).

Autoimmune thyroiditis is characterized by elevated peripheral pro-inflammatory biomarkers and a state of low-grade systemic chronic inflammation (6–9). While arterial dissection in patients with autoimmune thyroiditis and thyrotoxicosis has been documented in sparse case reports, the association remains inconclusive (10). Subsequent studies have suggested a potential involvement of thyroid autoimmunity in the pathogenesis of artery dissection; however, other investigations have reported inconsistent findings (11–14). A critical limitation of previous research is the inconsistent inclusion of participants with normal thyroid function. Furthermore, only a limited number of studies have specifically examined the relationship between thyroid antibodies and Spontaneous Cervicocranial Artery Dissection (SCCAD) (11, 13). Consequently, it remains unclear whether thyroid dysfunction or the presence of thyroid antibodies is the primary factor associated with SCCAD. The significant correlation between thyroid antibodies and endothelial dysfunction offers a plausible mechanism by which thyroid autoimmunity could contribute to the pathogenesis of dissections (15). Nevertheless, the potential influence of thyroid autoimmunity on the anatomical distribution of arterial dissections is currently unknown.

Therefore, we conducted a retrospective study to investigate the association between thyroid autoimmunity positivity, as well as its associated antibodies (TPOAb and TGAb), and SCCAD in euthyroid patients. This study also explored the interaction between thyroid autoimmunity and potential risk factors for SCCAD occurrence. Furthermore, we examined whether thyroid autoantibodies are associated with the anatomical distribution of arterial dissections (unilateral vs. bilateral, extracranial vs. intracranial).

## Materials and methods

### Study design and participants

We conducted a retrospective case-control study. Cases were consecutively recruited from patients diagnosed with SCCAD at the First Affiliated Hospital of Zhengzhou University between June 2021 and May 2025. Control subjects were randomly selected from individuals undergoing annual physical examinations at our hospital, with a case-to-control ratio of 1:1. All participants in both groups underwent cervical cerebrovascular ultrasonography to confirm the presence or absence of SCCAD. SCCAD was diagnosed based on the presence of local symptoms (e.g., neck pain, headache, or tinnitus) and/or signs of cerebral ischemia or subarachnoid hemorrhage. The diagnosis was definitively confirmed by digital subtraction angiography (DSA), which revealed characteristic features such as the string sign, pseudoaneurysm, intimal flap, double lumen, intramural hematoma, or underlying arteriopathy. Additionally, DSA findings were corroborated by at least one other imaging modality: high-resolution magnetic resonance imaging (HR-MRI), magnetic resonance angiography (MRA), computed tomography angiography (CTA), axial MRI of the neck, or cervical cerebrovascular ultrasonography. Exclusion criteria were defined as follows: (1) severe blunt head or neck trauma; (2) a history of iatrogenic cervicocranial artery injury or craniocervical vascular surgery within the preceding 6 months; (3) clinical or subclinical hyperthyroidism or hypothyroidism; and (4) prior use of anti-thyroid medications or thyroid hormone drugs.

This study was conducted in accordance with the Declaration of Helsinki and the ethical standards of the institutional or national research committee. The study protocol was approved by the ethics committee of the First Affiliated Hospital of Zhengzhou University (2025-KY-0922), and the need for obtaining patient informed consent was waived since all data were retrospectively collected and individual information was not disclosed.

### Data collection

Baseline data for all participants were obtained from electronic clinical records. The collected information included demographic characteristics (age and gender), medical history including smoking status, comorbidities (hypertension, diabetes, hyperlipidemia, hyperhomocysteinemia), and history of headache or neck pain, arterial dysplasia, recent infection (within 4 weeks), or trivial trauma and laboratory parameters [free triiodothyronine (FT3), free thyroxine (FT4), thyroid-stimulating hormone (TSH), anti-thyroid peroxidase antibody (TPOAb), and anti-thyroglobulin antibody (TGAb)]. For patients with SCCAD, the specific vascular segments involved in the dissection were also recorded.

### Assessment of thyroid autoantibody

Elevated thyroid autoantibodies were determined as either TPOAb > 34 U/mL or TGAb > 115 U/mL in accordance with the manufacturer's reference. The thyroid autoimmunity positivity was defined as patients had either elevated TPOAb or positive TGAb, or both.

### Assessment of angiography

The specific affected arteries in all SCCAD patients were assessed using comprehensive vascular imaging. Affected sites were categorized as follows: (1) Internal Carotid Artery (ICA) System: Segments were classified as C1 (cervical), C2 (petrous), C3 (lacerum), C4 (cavernous), C5 (clinoid), C6 (ophthalmic), C7 (communicating), MCA, and ACA. For analysis, the C1 segment was designated as extracranial, and segments C2 through C7, MCA, and ACA as intracranial. (2) Vertebral Artery (VA) System: Segments were classified as V1 (preforaminal), V2 (foraminal), V3 (suboccipital), and V4 (intracranial). The V4 segment was designated as intracranial, and segments V1 through V3 as extracranial.

### Statistical analysis

All statistical analyses were performed using IBM SPSS Statistics software (version 27). To describe the baseline characteristics, the continuous variables were expressed as mean and standard deviation (SD), and the categorical variables were expressed as frequency and percentage. To compare the difference between groups, a Student's *t* test was conducted for continuous data following the normal distribution, and a nonparametric test (Mann–Whitney *U* test) was conducted for the continuous data that did not follow a normal distribution. A Chi square test was conducted for categorized data. To minimize baseline differences and potential confounders between both groups, we performed 1:1 propensity score matching (PSM). The propensity score was estimated based on the following covariates: age, sex, comorbidities (hypertension, diabetes, hyperlipidemia, and hyperhomocysteinemia), arterial dysplasia, recent infection or trivial trauma (within 4 weeks), and thyroid function.

Univariate and multivariate logistic regressionanalyses were performed to explore whether thyroid autoimmunity positivity and elevated thyroid autoantibodies were associated with SCCAD. Because thyroid autoimmunity positivity, elevated TPOAb, and elevated TGAb are biologically correlated and entering them together could introduce multicollinearity, these three variables were entered into three separate multivariable logistic regression models. Variables with *P* values less than or equal to 0.1 in univariate analysis and factors with clinical significance were included in a further multivariate logistic analysis. This threshold was selected to reduce the risk of omitting clinically or biologically relevant confounders that might not achieve statistical significance at the conventional *P* < 0.05 level due to limited sample size. The odds ratio (OR) with 95% confidence interval (CI) was estimated to evaluate the effects.

Furthermore, stratified analyses and interaction tests were performed to evaluate whether the associations between thyroid autoimmunity (thyroid autoimunity positivity, elevated TPOAb, or elevated TGAb) and SCCAD incidence varied across patient subgroups. To assess effect heterogeneity, interaction test was assessed by introducing a product term (thyroid autoimmunity marker × subgroup) into a multivariate logistic regression model that contained the thyroid autoimmunity marker, subgroup variables and other covariates. The statistical significance of the interactions was assessed by the likelihood ratio test.

Finally, the SCCAD cohort was stratified into two groups: internal carotid artery dissections (ICDs) and vertebral artery dissection (VADs). The association between thyroid autoimmunity and dissection characteristics (unilateral vs. bilateral, intracranial vs. extracranial) was first assessed using the Chi-square test in these subgroups. Subsequently, multivariate logistic regression analyses were employed to further identify these relationships. Segment-specific analyses were performed at the level of the affected arterial segment (V3 or V4). Because a single patient could contribute multiple affected segments, these observations were not fully independent. Therefore, we further conducted patient-level sensitivity analyses (e.g., presence vs. absence of any V3 or V4 involvement) to confirm the robustness of the segment-level findings.

Probability values less than 0.05 on two-sided tests were considered significant in all statistical analyses.

## Results

### Characteristics of the study population

The study comprised 164 SCCAD patients and 164 matched controls. Among the patients, 79 (48.2%) had dissections in the internal carotid artery (ICA) system, 78 (47.6%) in the vertebral artery (VA) system, and 7 (4.2%) in both systems. Regarding laterality, a total of 171 dissection events were identified, of which the majority (149; 87.1%) were unilateral, 18 (10.5%) were bilateral, and 4 (2.4%) involved the basilar artery alone. In terms of the anatomical distribution, a total of 191 affected arterial segments were identified, of which 55 (28.8%) were extracranial, 98 (51.3%) were intracranial, and 38 (19.9%) involved both intracranial and extracranial segments. Of note, two patient had two non-contiguous dissections within the same vessel.

Baseline characteristics of the case and control groups are summarized in Table 1. Compared with controls, patients with SCCAD were significantly younger (*P* < 0.001), had a higher proportion of males (*P* < 0.001), and demonstrated a higher prevalence of migraine/neck pain (*P* < 0.001), current smoking (*P* = 0.032), recent infection (*P* = 0.030), and arterial dysplasia (*P* < 0.001). No significant differences were observed in hypertension, diabetes, hyperlipidemia, hyperhomocysteinemia, or thyroid function (TSH, FT3, FT4). Notably, the SCCAD group had a significantly higher prevalence of elevated TPOAb (*P* = 0.004), elevated TGAb (*P* = 0.004), and thyroid autoimmunity positivity (*P* < 0.001). After propensity score matching, no significant differences in baseline characteristics remained between SCCAD and healthy control groups.

**Table 1:** Baseline characteristics of patients with SCCAD and healthy controls before and after propensity score matching.

	Unmatched			Matched		
	SCCAD (*n* = 164)	Control (*n* = 164)	*P* value	SCCAD (*n* = 76)	Control (*n* = 76)	*P* value
Age, mean ± SD	46.15 ± 12.53	57.63 ± 9.61	<0.001	53.17 ± 11.82	51.11 ± 8.33	0.823
Male, *n* (%)	133 (81.1)	99 (60.4)	<0.001	53 (69.7)	45 (59.2)	0.175
Hypertension, *n* (%)	55 (33.5)	58 (35.4)	0.727	30 (39.5)	22 (28.9)	0.171
Diabetes, *n* (%)	15 (9.1)	24 (14.6)	0.125	10 (13.2)	8 (10.5)	0.616
Hyperlipidemia, *n* (%)	29 (17.7）	25 (15.2)	0.551	15 (19.7)	10 (13.2)	0.274
Hyperhomocysteinemia, *n* (%)	17 (10.4)	19 (11.6)	0.936	6 (7.9)	7 (9.2)	0.879
Headache/neck pain, *n* (%)	77 (47.0)	24 (14.6)	<0.001	24 (31.6)	12 (15.8)	0.022
Recent infection, *n* (%)	6 (3.7)	0	0.030	0	0	-
Trivial trauma, *n* (%)	3 (1.8)	0	0.248	0	0	-
Arterial dysplasia, *n* (%)	22 (13.4)	2 (1.2)	<0.001	2 (2.6)	2 (2.6)	1.000
Smoking			0.032			0.296
Former, *n* (%)	18 (11.0)	23 (14.0)		12 (15.8)	10 (13.2)	
Current, *n* (%)	55 (33.5)	34 (20.7)		21 (27.6)	14 (18.4)	
Never, *n* (%)	91 (55.5)	107 (65.2)		43 (56.6)	52 (68.4)	
TSH, mean ± SD	2.42 ± 1.73	2.64 ± 1.55	0.060	2.42 ± 1.57	2.49 ± 1.57	0.642
FT3, mean ± SD	4.38 ± 0.93	4.40 ± 1.38	0.266	4.40 ± 0.79	4.61 ± 1.52	0.742
FT4, mean ± SD	16.80 ± 2.96	17.18 ± 5.30	0.898	16.70 ± 3.00	17.32 ± 5.26	0.903
Thyroid autoimmunity positivity, *n* (%)	19 (11.6)	3 (1.8)	<0.001	9 (11.8)	2 (2.6)	0.028
Elevated TPOAb, *n* (%)	15 (9.1)	3 (1.8)	0.004	8 (10.5)	2 (2.6)	0.050
Elevated TGAb, *n* (%)	13(7.9)	2(1.2)	0.004	5(6.6)	1(1.3)	0.209

SCCAD, spontaneous cervicocranial arterial dissection; FT3, free triiodothyronine; FT4, free thyroxine; TSH, thyroid-stimulating hormone; TPOAb, thyroid peroxidase antibody; TGAb, thyroglobulin antibody.

### Thyroid autoimmunity and SCCAD

In the univariate analysis (Table 2), several factors showed significant associations with SCCAD. These included younger age (OR 0.913, 95% CI: 0.892–0.935, *P* < 0.001), male gender (OR 2.817, 95% CI: 1.707–4.648, *P* < 0.001), current smoking (OR 1.902, 95% CI: 1.141–3.170, *P* = 0.014), and arterial dysplasia (OR 12.549, 95% CI: 2.900–54.305, *P* < 0.001). Markers of thyroid autoimmunity were also strongly associated, including thyroid autoimmunity positivity (OR 7.032, 95% CI: 2.039–24.256, *P* = 0.002), elevated TPOAb (OR 5.403, 95% CI: 1.533–19.036, *P* = 0.009), and elevated TGAb (OR 6.974, 95% CI: 1.548–31.413, *P* = 0.011). In the multivariate analysis adjusted for potential confounders, thyroid autoimmunity positivity (OR 6.715; 95% CI: 1.589–28.377; *P* = 0.010) and elevated TPOAb (OR 5.999; 95% CI: 1.379–26.096; *P* = 0.017) remained independently associated with SCCAD. Age, gender, and arterial dysplasia were also independently associated with SCCAD. In contrast, elevated TGAb (OR 5.035; 95% CI: 0.924–27.433; *P* = 0.065) and current smoking lost their significant associations in the adjusted model (Table 3).

**Table 2:** Univariate Logistic regression analysis for factors associated with SCCAD.

	SCCAD	
	OR (95%CI)	*P* value
Age	0.913 (0.892–0.935)	<0.001
Male	2.817 (1.707–4.648)	<0.001
Hyperhomocysteinemia	0.895 (0.419–1.911)	0.774
Smoking		
Never	Ref	
Current	1.902 (1.141–3.170)	0.014
Former	0.920 (0.468–1.811)	0.810
Trivial trauma	-	0.999
Recent infection	-	0.999
Dysplasia	12.549 (2.900–54.305)	<0.001
Thyroid autoimmunity positivity	7.032 (2.039–24.256)	0.002
Elevated TPOAb	5.403 (1.533–19.036)	0.009
Elevated TGAb	6.974 (1.548–31.413)	0.011

SCCAD, spontaneous cervicocranial arterial dissection; TPOAb, thyroid peroxidase antibody; TGAb, thyroglobulin antibody.

**Table 3:** Multivariate logistic regression analysis for factors associated with SCCAD before matching.

	SCCAD, Model 1		SCCAD, Model 2		SCCAD, Model 3	
	OR (95% CI)	*P* value	OR (95% CI)	*P* value	OR (95% CI)	*P* value
Age	0.909 (0.885–0.934)	<0.001	0.908 (0.884–0.933)	<0.001	0.909 (0.885–0.934)	<0.001
Male	2.251 (1.138–4.454)	0.020	2.218 (1.126–4.370)	0.021	2.227 (1.133–4.380)	0.020
Hcy	0.830 (0.317–2.171)	0.704	0.804 (0.308–2.101)	0.657	0.773 (0.297–2.011)	0.598
Smoking						
Never	Ref		Ref		Ref	
Current	1.783 (0.907–3.506)	0.094	1.768 (0.900–3.475)	0.098	1.727 (0.882–3.383)	0.111
Former	1.907 (0.792–4.596)	0.150	1.899 (0.787–4.581)	0.154	1.913 (0.798–4.581)	0.146
Trivial trauma	-	0.999	-	0.999	-	0.999
Recent infection	-	0.999	-	0.999	-	0.999
Dysplasia	11.449 (2.260–57.998)	0.003	11.511 (2.285–57.978)	0.003	12.459 (2.462–63.043)	0.002
TAP	6.715 (1.589–28.377)	0.010	-	-	-	-
Elevated TPOAb	-	-	5.999 (1.379–26.096)	0.017	-	-
Elevated TGAb	-	-	-	-	5.035 (0.924–27.433)	0.062

Model 1: adjustment for age, gender, Hcy, smoking, trivial trauma, recent infection, dysplasia and TAP; Model 2: adjustment for age, gender, Hcy, smoking, trivial trauma, recent infection, dysplasia and TPOAb; Model 3: adjustment for age, gender, Hcy, smoking, trivial trauma, recent infection, dysplasia and TGAb.

SCCAD, spontaneous cervicocranial arterial dissection; Hcy, Hyperhomocysteinemia; TAP, thyroid autoimmunity positivity; TPOAb, thyroid peroxidase antibody; TGAb, thyroglobulin antibody.

**Figure 1:**
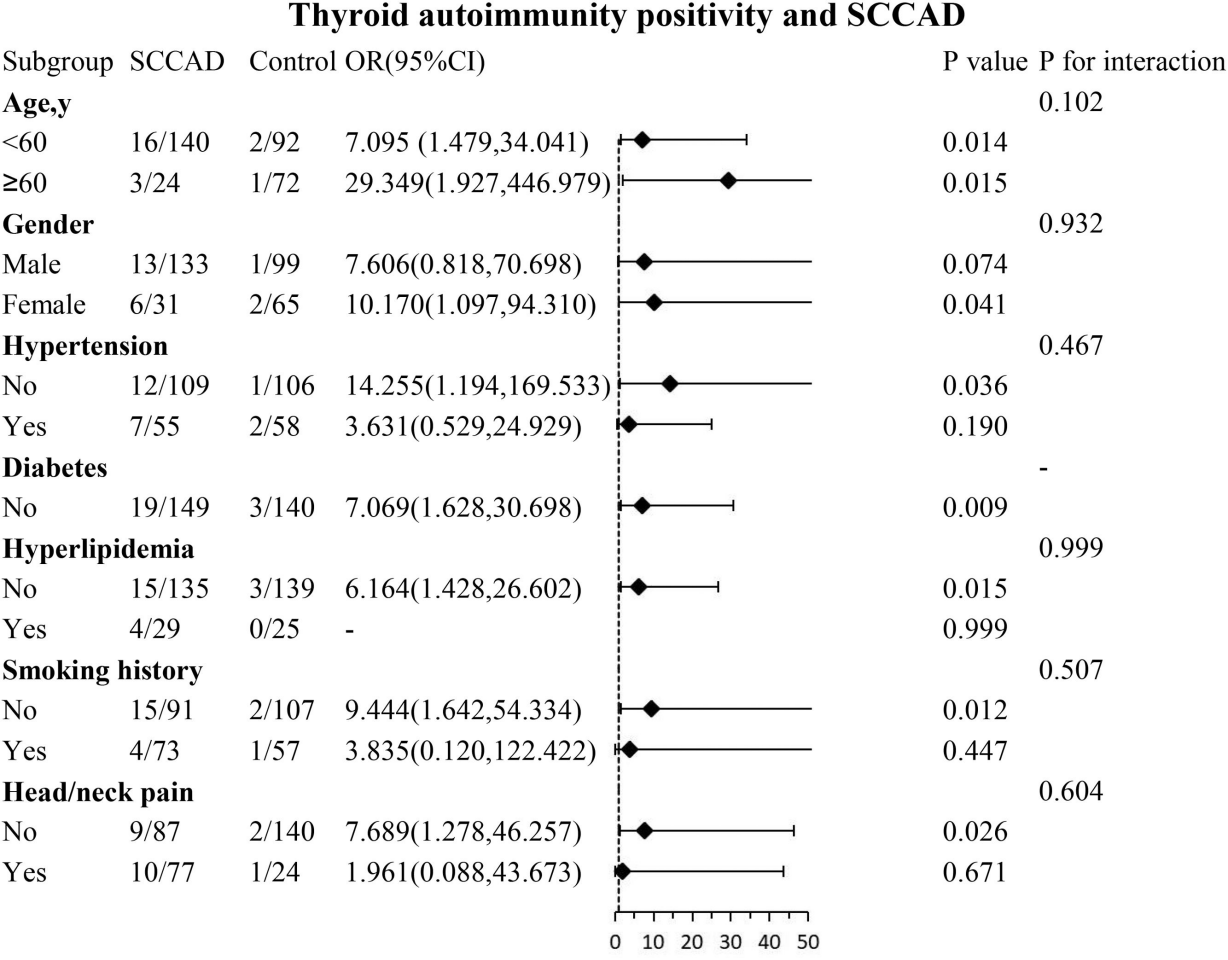
Forest plot of subgroup analyses and interaction tests for the association between thyroid autoimmunity positivity and SCCAD. Interaction *P*-values were calculated by including interaction terms (thyroid autoimmunity marker × subgroup variable) in multivariate logistic regression models. Estimates were adjusted for age, gender, smoking history, hyperhomocysteinemia, recent infection, trivial trauma, arterial dysplasia, thyroid autoimmunity markers (TPOAb, TGAb or thyroid autoimmunity positivity) and some subgroup variables (hypertension, diabetes, hyperlipemia, head/neck pain). The interaction *P* value was >0.05, indicating that the subgroup variables in the association between thyroid autoimmunity markers and SCCAD did not reach statistical significance.

**Figure 2:**
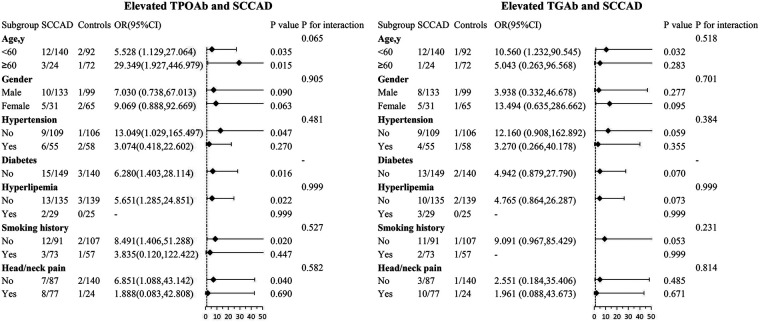
Forest plot of subgroup analyses and interaction tests for the association between thyroid autoantibodies and SCCAD. Interaction *P*-values were calculated by including interaction terms (thyroid autoimmunity marker × subgroup variable) in multivariate logistic regression models. Estimates were adjusted for age, gender, smoking status, hyperhomocysteinemia, recent infection, trivial trauma, arterial dysplasia, thyroid autoimmunity markers (TPOAb, TGAb or thyroid autoimmunity positivity) and some subgroup variables (hypertension, diabetes, hyperlipemia, head/neck pain). The interaction *P* value was >0.05, indicating that the subgroup variables in the association between thyroid autoimmunity markers and SCCAD did not reach statistical significance.

Further stratified analyses and interaction tests were performed to evaluate the influence of thyroid autoimmunity on SCCAD (Figures 1, 2). Thyroid autoimmunity positivity and elevated TPOAb were consistently positively associated with SCCAD across all subgroups, including each age subgroup and patients without hypertension, diabetes, hyperlipidemia, smoking history or head/neck pain. However, this association was not observed in male patients. Except for the younger age subgroup, elevated TPOAb was not associated with SCCAD in any of the other subgroups. Despite the lack of statistical significance in some subgroups, a consistent trend toward higher prevalence of thyroid autoimmunity was observed across these SCCAD subgroups. The consistent associations observed after the interaction tests (all *P* for interaction > 0.05) indicate the consistency of our primary findings regarding the relationship between thyroid autoimmunity and SCCAD.

### Thyroid autoimmunity and arterial dissections distribution

To explore the impact of thyroid autoimmunity on distribution of dissections, we divided SCCAD patients into two subgroups of different affected circulation systems (internal carotid artery dissections, ICDs; and vertebral artery dissections, VADs). Regarding ICDs, no statistically significant differences were observed in the prevalence of autoimmune thyroiditis or elevated thyroid autoantibody levels between unilateral and bilateral involvement, or between intracranial and extracranial distributions (Supplementary Table S1). As for the VADs, thyroid autoimmunity showed no significant association with unilateral, bilateral, or isolated basilar artery dissection; however, it was significantly correlated with both extracranial and intracranial involvement. More importantly, elevated TPOAb was significantly associated with VADs involving both the V4 (*P* = 0.016) and V3 (*P* = 0.028) segments (Supplementary Table S2).

### Segment-specific analysis of VADs with V3 and V4 involvement

Baseline characteristics of vertebral artery dissections with V3 and V4 segment involvement are summarized in the Table 4. Compared with dissections without V3 involvement, those with V3 involvement were significantly younger (40.43 ± 9.61 vs. 46.92 ± 10.88 years, *P* = 0.002) and had a higher proportion of male sex (87.0% vs. 68.0%, *P* = 0.027). Hypertension was less common in the V3 group (21.7% vs. 42.0%, *P* = 0.034), while arterial dysplasia was more frequently observed (30.4% vs. 12.0%, *P* = 0.026). No significant differences were found between the two groups regarding diabetes, hyperlipidemia, hyperhomocysteinemia, headache/neck pain, recent infection, trivial trauma, or smoking status (all *P* > 0.05). Regarding thyroid function parameters, FT4 levels were significantly lower in the V3 group (16.46 ± 2.31 vs. 17.78 ± 3.33, *P* = 0.028), whereas TSH and FT3 levels were comparable between groups (both *P* > 0.05). Thyroid autoimmunity positivity showed a borderline difference (6.5% vs. 20.0%, *P* = 0.054), and elevated TPOAb was significantly less frequent in the V3 group (2.2% vs. 18.0%, *P* = 0.028), while elevated TGAb did not differ significantly (*P* = 0.717). In contrast, no significant differences were observed between dissections with and without V4 involvement across all demographic, clinical, and laboratory variables, except for elevated TPOAb, which was significantly more prevalent in the V4 group (21.1% vs. 3.4%, *P* = 0.016). Thyroid autoimmunity positivity showed a borderline association with V4 involvement (21.1% vs. 8.6%, *P* = 0.082).

**Table 4:** Dissection-level baseline characteristics of vertebral artery dissections involving vs. sparing the V3 and V4 segment.

	V3 (*n* = 46)	Non V3 (*n* = 50)	*P* value	V4 (*n* = 38)	Non V4 (*n* = 58)	*P* value
Age, mean ± SD	40.43 ± 9.61	46.92 ± 10.88	0.002	45.68 ± 9.40	42.59 ± 11.44	0.164
Male, *n* (%)	40 (87.0)	34 (68.0)	0.027	28 (73.7)	46 (79.3)	0.521
Hypertension, *n* (%)	10 (21.7)	21 (42.0)	0.034	13 (34.2)	18 (31.0)	0.745
Diabetes, *n* (%)	4 (8.7)	8 (16.0)	0.280	3 (7.9)	9 (15.5)	0.430
Hyperlipidemia, *n* (%)	11 (23.9）	6 (12.0)	0.127	6 (15.8)	11 (19.0)	0.690
Hyperhomocysteinemia, *n* (%)	4 (8.7)	5 (10.0)	0.953	4 (10.5)	5 (8.6)	0.951
Headache/neck pain, *n* (%)	32 (69.6)	27 (54.0)	0.117	24 (63.2)	35 (60.3)	0.782
Recent infection, *n* (%)	3 (6.5)	0	0.106	0	3 (5.2)	0.275
Trivial trauma, *n* (%)	0	1 (2.0)	1.000	1 (2.6)	0	0.396
Arterial dysplasia, *n* (%)	14 (30.4)	6 (12.0)	0.026	11 (28.9)	9 (15.5)	0.113
Smoking			0.233			0.868
Former, *n* (%)	3 (6.5)	6 (12.0)		3 (7.9)	6 (10.3)	
Current, *n* (%)	17 (37.0)	11 (22.0)		12 (31.6)	16 (27.6)	
Never, *n* (%)	26 (56.5)	33 (66.0)		23 (60.5)	36 (62.1)	
TSH, mean ± SD	2.09 ± 1.50	2.29 ± 1.66	0.577	2.02 ± 1.52	2.30 ± 1.62	0.361
FT3, mean ± SD	4.08 ± 0.82	4.31 ± 0.78	0.108	4.05 ± 0.90	4.30 ± 0.73	0.205
FT4, mean ± SD	16.46 ± 2.31	17.78 ± 3.33	0.028	16.80 ± 3.04	17.38 ± 2.89	0.857
Thyroid autoimmunity positivity, *n* (%)	3 (6.5)	10 (20.0)	0.054	8 (21.1)	5 (8.6)	0.082
Elevated TPOAb, *n* (%)	1 (2.2)	9 (18.0)	0.028	8 (21.1)	2 (3.4)	0.016
Elevated TGAb, *n* (%)	3(6.5)	5(10.0)	0.717	4(10.5)	4(6.9)	0.708

TSH, thyroid-stimulating hormone; FT3, free triiodothyronine; FT4, free thyroxine; TPOAb, thyroid peroxidase antibody; TGAb, thyroglobulin antibody.

After multivariate adjustment, arterial dysplasia remained significantly associated with VADs involving V3 segment (OR 6.595, 95% CI: 1.492–29.152, *P* = 0.013). In contrast, elevated TPOAb levels were inversely associated with V3 involvement (OR 0.065, 95% CI: 0.005–0.816, *P* = 0.034, Table 5), but positively associated with V4 involvement (OR 7.663, 95% CI: 1.443–40.680, *P* = 0.017) (Table 5).

**Table 5:** Multivariate analysis of factors associated with V3/V4 involvement vs. non-V3/V4 vertebral artery dissections (segment-level).

	V3 involvement		V4 involvement	
	OR (95% CI)	*P* value	OR (95% CI)	*P* value
Age	0.944 (0.890–1.003)	0.061	1.027 (0.978–1.078)	0.282
Male	2.517 (0.626–10.126)	0.194	0.878 (0.255–3.021)	0.836
Hypertension	0.301 (0.078–1.155)	0.080	–	–
Dysplasia	6.595 (1.492–29.152)	0.013	2.667 (0.861–8.255)	0.089
FT4	0.843 (0.697–1.020)	0.079	–	–
Elevated TPOAb	0.065 (0.005–0.816)	0.034	7.663 (1.443–40.680)	0.017

Multivariate analysis for V3: adjustment for age, gender, hypertension, hyperhomocysteinemia, smoking, trivial trauma, recent infection, dysplasia, FT4 and TPOAb; Multivariate analysis for V4: adjustment for age, gender, hyperhomocysteinemia, smoking, trivial trauma, recent infection, dysplasia and TPOAb. FT4, free thyroxine; TPOAb, thyroid peroxidase antibody.

### Patient-level sensitivity analyses

A patient-level sensitivity analysis was performed in 85 patients (V3: *n* = 44, non-V3: *n* = 41; V4: *n* = 37, non-V4: *n* = 48; Supplementary Table S3). As shown in Supplementary Table S3, patients with V3 involvement were significantly younger (*P* = 0.004), more often male (*P* = 0.006), less likely to have hypertension (*P* = 0.038), and more likely to have arterial dysplasia (*P* = 0.013) compared with those without V3 involvement. Elevated TPOAb was significantly less frequent in the V3 group (2.3% vs. 17.1%, *P* = 0.026). In contrast, V4 involvement was only associated with a higher prevalence of elevated TPOAb (18.9% vs. 2.1%, *P* = 0.019), with no other significant differences observed (all *P* > 0.05).

In the multivariate analysis, arterial dysplasia was associated with V3 involvement (OR 10.323, 95% CI: 1.933–55.115, *P* = 0.006), whereas elevated TPOAb showed an inverse correlation with V3 involvement (OR 0.043, 95% CI: 0.003–0.669, *P* = 0.025). For V4 involvement, elevated TPOAb was the sole factor showing a significant correlation (OR 10.452, 95% CI: 1.143–95.584, *P* = 0.038), while arterial dysplasia yielded only a borderline association (*P* = 0.092).

## Discussion

This study investigated the association between thyroid autoimmunity and spontaneous cervicocranial artery dissection (SCCAD) in euthyroid patients. Our results revealed a significantly higher prevalence of thyroid autoimmunity positivity and elevated anti-thyroid peroxidase antibody (TPOAb) levels in the SCCAD group, with consistent associations observed across various subgroups in interaction analyses. Notably, elevated TPOAb levels were specifically associated with dissections involving the V3 and V4 segments of the vertebral artery.

The presence of inflammatory infiltrates in the arterial wall of dissections, as evidenced by pathology and imaging, implies that localized inflammatory changes might be a crucial for the development of spontaneous carotid artery dissection (3–5). Previous results demonstrated that inflammatory markers were elevated in patients with cervical artery dissection (1, 2, 16). Moreover, two reports also indicated that arterial wall inflammation was associated with spontaneous vertical artery dissection, and treatment with aspirin/steroids resulted in significant improvement in both clinical manifestations and brain lesions detected by magnetic resonance imaging (17, 18). Given the details above, the activation of specific immune-mediated mechanisms may be responsible for local inflammatory alterations associated with SCCAD.

Notably, in the present study, SCCAD was associated with a higher presence of thyroid autoimmunity and thyroid autoantibody levels, particularly TPOAb. This association was further confirmed by propensity score matching analysis, albeit with borderline statistical significance in TPOAb level. After adjusting for potential risk factors, thyroid autoimmunity positivity and TPOAb levels remained significantly associated with SCCAD, indicating a robust and independent relationships. The results in our study are consistent with one previous study about thyroid autoimmunity and spontaneous cervical artery dissection (11). Our findings are also in accordance with the study by Li, in which they found that a higher ratio of autoimmune thyroid diseases was observed in SCCAD patients (12). Similarly, previous study has shown that cytokines produced by T cells in autoimmune thyroiditis can stimulate the production of nitric oxide and prostaglandins, thereby enhancing the inflammatory response in autoimmune thyroiditis (19). This notion also supports our finding that thyroid autoimmunity may contribute to the development of SCCAD. Furthermore, elevated thyroid autoantibodies has been reported to correlate moyamoya disease, suggesting the role of thyroid autoantibodies in immune-mediated vascular disease (20, 21). Nevertheless, some study have failed to establish a role for thyroid autoimmunity in cervical artery dissection pathogenesis (13). This discrepancy likely stems from a key methodological limitation in the earlier work: the absence of a dedicated control group. Specifically, that study compared the incidence of thyroid autoimmunity in the SCCAD cohort against historical data from population-based studies. This approach is compromised by significant potential bias arising from heterogeneity in participant selection and methodological inconsistencies.

Interestingly, we found that there likely was a higher percentage of males in SCCAD patients when compared with the healthy control group. After adjusting the confounding factors, the association between males and SCCAD remained unchanged. In population-based studies of SCCAD, there appears to be a slight gender predisposition favoring males (22, 23), which were similar to our results. According to our knowledge, most autoimmune diseases are more prevalent in females than in males, because estrogens are potent stimulators of autoimmunity (24). The gender predilection for more males in SCCAD observed in our study may be explained by the protective influence of estrogen on the cerebral vasculature. Moreover, our results showed a higher percentage of thyroid autoimmunity positivity and elelvated thyroid autoantibodies (TPOAb and TGAb) in female SCCAD compared with that in male SCCAD patients (thyroid autoimmunity positivity:19.4% vs. 9.8%; positive TPOAb: 16.1% vs. 7.5%; TGAb: 16.1% vs. 6.0%), which agreed with the statement that autoimmune diseases are more prevalent in females. We also observed an inverse association between age and SCCAD, corroborating the established evidence that this condition predominantly affects younger individuals, with a mean age of approximately 45 years as reported in previous studies (25–28). In addition, consistent with previous reports, arterial dysplasia was found to be associated with SCCAD (29–31).

Regarding the laterality of SCCAD (unilateral vs. bilateral), we found no significant differences in the prevalence of elevated thyroid autoantibody levels (TGAb and TPOAb) or thyroid autoimmunity positivity between the involved arterial systems (vertebral and corotid arteries). Although evidence regarding the role of thyroid autoimmunity in unilateral vs. bilateral arterial lesions remains limited, one prior study notably reported a comparable association between elevated TPOAb levels and cerebral arterial stenotic lesions (32).

Intriguingly, our analysis revealed a segment-dependent association between elevated TPOAb and VADs, showing a positive association at the V4 segment and an inverse association at the V3 segment. Conversely, elevated TGAb exerted no significant independent influence on the intracranial or extracranial distribution of VADs. Despite an unelucidated mechanism, this association is supported by the recognized role of immune responses in SCCAD. In autoimmune thyroiditis with euthyroidism, vascular studies of the brachial artery showed that endothelium-mediated arterial dilation was negatively correlated with TPOAb, which suggests that elevated TPOAb may cause endothelial dysfunction (15). Since endothelial dysfunction is an important early event in SCCAD, further investigations may be needed to clarify the relationship between TPOAb and endothelial dysfunction in SCCAD patients. In autoimmune thyroiditis, unlike TGAb, a higher TPOAb level was associated with a significantly increased frequency of T cells producing interferon (IFN)-γ compared to healthy donors, thereby promoting inflammatory alterations through the autoimmune response (33, 34). In accordance with our findings, elevated TPOAb, but not elevated TGAb, has been independently associated with intracranial arterial stenosis in stroke patients (35, 36). This contrasts with a study by Tanaka et al., which reported that both TPOAb and TGAb were significantly linked to stenotic lesions in the terminal internal carotid artery (32). Crucially, by employing multivariate logistic regression to adjust for potential confounders, our study enabled a more robust assessment of the independent role of thyroid autoantibodies in specific affected arterial segments.

The present study has several methodologic strengths. Given the infrequent occurrence of sVAD, a hospital-based, case-control design is feasible for the investigation of associations between thyroid autoimmunity and SCCAD while controlling for important confounding factors. In addition, stratified analyses by subgroups further evaluated the associations between thyroid autoimmunity and SCCAD, and the interaction analyses yielded *P*-values >0.05 for all subgroups, indicating no significant effect modification and thereby strengthening the credibility of our primary findings.

Several limitations of this study need to be acknowledged. First, the retrospective design and the relatively limited sample size might introduce bias, and thus the results should be interpreted cautiously. Second, although rigorous methods were used for SCCAD detection, the inherent limitations of retrospective study may have led to some missed diagnosis and misdiagnosis. Third, the study population comprised ethnically Chinese patients from a single center, so the observed prevalence of thyroid autoimmunity may not be generalizable to other populations. Fourth, the use of hospital-based healthy controls may have introduced selection bias, as these individuals may not be fully representative of the general population. Fifth, as in all case-control studies, the causality of the association between thyroid autoimmunity and spontaneous cervical artery dissections could not be assessed. Sixth, despite multivariate adjustment for several potential confounders, residual confounding due to unmeasured or imperfectly measured factors cannot be entirely excluded. Seventh, the 1:1 matching design may have been insufficient to fully balance age and sex between groups and a recommendation for future studies to adopt a 1:2 (or higher) matching ratio and to apply stricter age matching and more rigorous sex matching to minimize confounding. Eighth, the limited sample size, particularly after stratifying by vertebral artery segments (V3 vs. V4), reduced the statistical power of our segment-specific analyses. Therefore, these results should be interpreted as exploratory rather than confirmatory, and all findings require validation in larger independent cohorts. Ninth, our current study adopted only a binary classification of antibody positivity, which may not fully capture the dose-dependent effects of antibody levels. Therefore, we will recommend that future investigations incorporate TPOAb and TgAb titers as continuous covariates in multivariable logistic regression models, so as to more rigorously elucidate the potential dose-response relationship between thyroid autoantibodies and SCCAD. Finally, although patient-level sensitivity analyses were performed to address within-patient correlation, we cannot completely exclude residual confounding due to unmeasured patient-level factors. For these reasons, our findings should be considered hypothesis-generating, and replication in larger, prospective, multicenter cohorts with segment-level data is warranted.

In conclusion, thyroid autoimmunity positivity and elevated TPOAb levels were independently associated with SCCAD. More importantly, we identified a previously unrecognized segment-specific duality of TPOAb in vertebral artery dissections, with divergent ralationships at the V3 and V4 segments These findings raise the possibility that thyroid autoimmunity may be involved in the local inflammatory cascade predisposing to arterial dissection Given the exploratory nature of our observations, further prospective studies with larger sample sizes are required to corroborate these results and comprehensively delineate the mechanistic pathways involved.

## Data Availability

The raw data supporting the conclusions of this article will be made available by the authors, without undue reservation.
