# Supplementary material for: Thyroid autoimmunity and spontaneous cervicocranial artery dissection: an exploratory segment-specific case-control study
Source: Front Cardiovasc Med. 2026 Jul 15;13:1733516. doi: 10.3389/fcvm.2026.1733516 (PMC13416261; doi:10.3389/fcvm.2026.1733516)
Supplement: Supplementary file 1 [file Table1.docx]

Table 1S. Association of ICDs with TAP, TPOAb, and TGAb.

|  | Total N | TAP n(%) | P value | TPOAb (+)  n (%) | P value | TGAb (+)  n (%) | P value |
| --- | --- | --- | --- | --- | --- | --- | --- |
| Bilateral | 8 | 1 (12.5) | 0.558 | 1 (12.5) | 0.508 | 1 (12.5) | 0.454 |
| Unilateral | 78 | 7 (9.0) |  | 6 (7.7) |  | 5 (6.4) |  |
| Intracranial | 26 | 0 (0) | 0.094 | 0 (0) | 0.118 | 0 (0) | 0.218 |
| Extracranial | 44 | 5 (11.4) |  | 4 (9.1) |  | 4 (9.1) |  |
| Extra+intracranial | 25 | 4 (16.0) |  | 4 (16.0) |  | 3 (12.0) |  |

Abbreviations: P values were derived from the chi-square test or Fisher's exact test, as appropriate. SCCAD, spontaneous cervicocranial arterial dissection; TAP, thyroid autoimmunity positivity; TPOAb, thyroid peroxidase antibody; TGAb, thyroglobulin antibody; ICDs, internal carotid artery dissections.

Table 2S. Association of VADs with TAP, TPOAb, and TGAb

|  | Total N | TAP n(%) | P value | TPOAb (+)  n (%) | P value | TGAb (+)  n (%) | P value |
| --- | --- | --- | --- | --- | --- | --- | --- |
| Bilateral | 10 | 2 (20.0) | 0.367 | 2 (20.0) | 0.139 | 1 (10.0) | 1.000 |
| Unilateral | 71 | 8 (11.3) |  | 5 (7.0) |  | 6 (8.5) |  |
| Basilar | 4 | 1 (25.0) |  | 1 (25.0) |  | 0 (0) |  |
| Extracranial | 54 | 4 (7.4) | **0.005** | 1 (1.9) | **<0.001** | 4 (7.4) | 0.388 |
| Intracranial | 29 | 9 (31.0) |  | 9 (31.0) |  | 4 (13.8) |  |
| Extra+intracranial | 13 | 0 (0) |  | 0 (0) |  | 0 (0) |  |
| V1segment | 19 | 0 (0) | 0.121 | 0 (0) | 0.215 | 0 (0) | 0.315 |
| V2segment | 28 | 3 (10.7) | 0.848 | 0 (0) | 0.076 | 3 (10.7) | 0.892 |
| V3segment | 46 | 3 (6.5) | 0.054 | 1 (2.2) | **0.028** | 3 (6.5) | 0.717 |
| V4segment | 38 | 8 (21.1) | 0.082 | 8 (21.1) | **0.016** | 4 (10.5) | 0.708 |
| Basilar | 5 | 1 (20.0) | 0.525 | 1 (20.0) | 0.430 | 0 (0) | 1.000 |

Abbreviations: P values were derived from the chi-square test or Fisher's exact test, as appropriate. Bold values indicate P < 0.05, considered statistically significant. SCCAD, spontaneous cervicocranial arterial dissection; TAP, thyroid autoimmunity positivity; TPOAb, thyroid peroxidase antibody; TGAb, thyroglobulin antibody; ICDs, internal carotid artery dissections; VADs, vertebral artery dissections.

Table 3S Patient-level baseline characteristics of vertebral artery dissections involving versus sparing the V3 and V4 segment

|  | V3  (n=44) | Non V3  (n=41) | P value |  | V4  (n=37) | Non V4  (n=48) | P value |
| --- | --- | --- | --- | --- | --- | --- | --- |
| Age,mean±SD | 40.59±9.79 | 47.66±11.68 | 0.004 |  | 45.95±9.39 | 42.50±12.39 | 0.120 |
| Male,n(%) | 39(88.6) | 26(63.4) | 0.006 |  | 27(73.0) | 38(79.2) | 0.504 |
| Hypertension,n(%) | 10(22.7) | 18(43.9) | 0.038 |  | 13(35.1) | 15(31.3) | 0.706 |
| Diabetes,n(%) | 4(9.1) | 8(19.5) | 0.168 |  | 3(8.1) | 9(18.8) | 0.162 |
| Hyperlipidemia,n(%) | 11(25.0） | 4(9.8) | 0.065 |  | 6(16.2) | 9(18.8) | 0.761 |
| Hyperhomocysteinemia,n(%) | 4(9.1) | 5(12.2) | 0.769 |  | 4(10.8) | 5(10.4) | 0.989 |
| Headache/neck pain,n(%) | 30(68.2) | 20(48.8) | 0.069 |  | 24(64.9) | 26(54.2) | 0.320 |
| Recent infection,n(%) | 3(6.8) | 0 | 0.242 |  | 0 | 3(6.3) | 0.254 |
| Trivial trauma,n(%) | 0 | 1(2.4) | 0.482 |  | 1(2.7) | 0 | 0.435 |
| Arterial dysplasia,n(%) | 14(31.8) | 4(9.8) | 0.013 |  | 11(29.7) | 7(14.6) | 0.090 |
| Smoking |  |  | 0.148 |  |  |  | 0.797 |
| Former,n(%) | 3(6.8) | 6(14.6) |  |  | 3(8.1) | 6(12.5) |  |
| Current,n(%) | 16(36.4) | 8(19.5) |  |  | 11(29.7) | 13(27.1) |  |
| Never,n(%) | 25(56.8) | 27(65.9) |  |  | 23(62.2) | 29(60.4) |  |
| TSH,mean±SD | 2.10±1.51 | 2.36±1.58 | 0.377 |  | 2.07±1.51 | 2.34±1.57 | 0.343 |
| FT3,mean±SD | 4.13±0.80 | 4.29±0.83 | 0.257 |  | 4.05±0.91 | 4.32±0.72 | 0.213 |
| FT4,mean±SD | 16.59±2.27 | 17.34±3.21 | 0.213 |  | 16.71±3.03 | 17.13±2.57 | 0.972 |
| Thyroid autoimmunity positivity,n(%) | 3(6.8) | 8(19.5) | 0.081 |  | 7(18.9) | 4(8.3) | 0.265 |
| Elevated TPOAb,n(%) | 1(2.3) | 7(17.1) | 0.026 |  | 7(18.9) | 1(2.1) | 0.019 |
| Elevated TGAb,n(%) | 3(6.8) | 4(9.8) | 0.707 |  | 4(10.8) | 3(6.3) | 0.694 |

Abbreviations: TSH, thyroid-stimulating hormone; FT3, free triiodothyronine; FT4, free thyroxine; TPOAb, thyroid peroxidase antibody; TGAb, thyroglobulin antibody.

Table 4S. Multivariate analysis of factors associated with V3/V4 involvement versus non-V3/V4 vertebral artery dissections (patient-level)

|  | V3 involvement  OR (95%CI), P value | | V4 involvement  OR (95%CI), P value | |
| --- | --- | --- | --- | --- |
| Age | 0.958(0.903-1.016) | 0.153 | 1.028(0.979-1.079) | 0.270 |
| Male | 4.577(0.984-21.287) | 0.052 | 1.135(0.308-4.186) | 0.849 |
| Hypertension | 0.291(0.067-1.260) | 0.099 | - | - |
| Dysplasia | 10.323(1.933-55.115) | 0.006 | 2.809(0.844-9.354) | 0.092 |
| Elevated TPOAb | 0.043(0.003-0.669) | 0.025 | 10.452(1.143-95.584) | 0.038 |

Multivariate analysis for V3: adjustment for age, gender, hypertension, hyperlipidemia, hyperhomocysteinemia, smoking, trivial trauma, recent infection, dysplasia and TPOAb; Multivariate analysis for V4: adjustment for age, gender, hyperhomocysteinemia, smoking, trivial trauma, recent infection, dysplasia and TPOAb. Abbreviations: TPOAb, thyroid peroxidase antibody.
